# Precision targeting of genetic variations in mixed bacterial cultures using CRISPR-Cas12a-programmed λ phages

**DOI:** 10.3389/fmicb.2025.1575339

**Published:** 2025-06-02

**Authors:** Chan Kyeong Lee, Ho Joung Lee, Song Hee Jeong, Sang Jun Lee

**Affiliations:** Department of Systems Biotechnology, Institute of Microbiomics, Chung-Ang University, Anseong, Republic of Korea

**Keywords:** CRISPR-Cas12a, bacteriophage, lambda, lysogen, *Escherichia coli*

## Abstract

The CRISPR-Cas system, an adaptive immune mechanism in prokaryotes against bacteriophages, has been developed into a versatile tool for recognizing and cleaving target nucleic acid sequences. In this study, we developed a model system by integrating CRISPR-Cas12a into the genome of temperate bacteriophage λ, enabling precise regulation of lysogeny and lysis in *Escherichia coli*. We confirmed that λ phage, armed with Cas12a nuclease and CRISPR RNA (crRNA) targeting specific sequences, could inhibit the lysogenic cycle of *E. coli* cells. We demonstrated that the CRISPR-Cas12a-loaded temperate λ phage mimicked a lytic phage by selectively killing cells carrying the target genomic sequence. Furthermore, by employing truncated crRNA to enhance target recognition specificity, we found that the synthetic phage could distinguish single nucleotide variations in the genomic target DNA, enabling precise targeting and selective elimination of target cells in homogeneous bacterial cultures. To further validate its specificity, we tested this system in mixed bacterial cultures, wherein Cas12a nuclease and truncated crRNA-loaded bacteriophages selectively eliminated only those cells carrying the target sequences perfectly matching the crRNA. These results highlight the potential of this approach for advancing precision microbiome modulation.

## 1 Introduction

The CRISPR-Cas system is an adaptive immune mechanism found in bacteria and archaea that protects the host from invading foreign genetic elements (Barrangou et al., 2007; Datsenko et al., 2012). Among these systems, the Type I-E CRISPR-Cas system in *Escherichia coli* is particularly well-characterized and has served as a model for studying CRISPR-Cas-mediated immunity (Makarova et al., 2011). The CRISPR-Cas system comprises a guide RNA (gRNA) that recognizes specific DNA sequences and a CRISPR-associated (Cas) nuclease that causes cleavage. By modifying the target recognition sequence (TRS) in the gRNA, various DNA targets can be specifically recognized and cleaved (Wiles et al., 2015). This system is also used for genome editing in various organisms, including microbes (Shen et al., 2019), plants (Li et al., 2020), animals (Niu et al., 2014), and humans (Gillmore et al., 2021).

Since the first demonstration of the genome editing of T7 bacteriophage using the CRISPR-Cas system, this system has evolved into an effective tool for bacteriophage genome engineering (Kiro et al., 2014). Among the various CRISPR systems, CRISPR-Cas9 has been widely used for bacteriophage genome editing owing to its high specificity and efficiency. For instance, Martel et al. utilized CRISPR-Cas9 to generate small and large deletions and point mutations and performed gene replacements in the *orf* of virulent phage 2972 (Martel and Moineau, 2014). Furthermore, CRISPR-Cas9 has been applied to edit the *cI^857^* gene of the λ phage at a single-nucleotide level, enabling its conversion to *cI^WT^* (Lee et al., 2022a). Recently, CRISPR-Cas12a has emerged as a promising alternative to Cas9 for genome editing in view of its lesser off-target effects, simplified gRNA design, and ease of cellular delivery (Ha et al., 2020; Lee et al., 2018; Slaman et al., 2024). Unlike Cas9, Cas12a generates staggered DNA ends upon cleavage, which promotes homology-directed repair and enhances the efficiency of transgene integration (Huang et al., 2022; Moescheid et al., 2023). Additionally, under specific conditions, Cas12a exhibits higher cleavage efficiency than Cas9, which makes it useful for bacteriophage genome editing (Chen et al., 2024; Dong et al., 2021).

As the CRISPR-Cas technology has facilitated bacteriophage genome engineering, its applications have expanded to encompass gene function identification, bacteriophage–host interactions, and pathogen detection. For example, Tao et al. employed CRISPR-Cas9 to delete the *rnlB* gene or introduce an amber mutation in T4 phage, demonstrating that *rnlB* is not essential for phage infection (Tao et al., 2017). Similarly, Hoshiga et al. (2019) replaced the tail fiber of T2 phage with that of PP01 phage using CRISPR-Cas9, enhancing its infectivity toward *E. coli* O157:H7 and demonstrating the critical role of the short tail fiber of PP01 in bacterial adsorption. Moreover, reporter phages carrying the nanoluciferase (*nluc*) gene have been developed for detecting urinary tract infections in clinical samples (Meile et al., 2023).

Originally a prokaryotic defense mechanism against bacteriophages, the CRISPR-Cas system has been repurposed to selectively eliminate specific microorganisms based on their DNA sequences via bacteriophage-mediated delivery (Balcha and Neja, 2023). The CRISPR-Cas system can be introduced into target cells via phagemids or phage genomes and has been used to eradicate harmful bacteria carrying antibiotic resistance or virulence genes (Fagen et al., 2017). For instance, a CRISPR-Cas9 system targeting the β-lactam resistance genes *bla_NDM-1_* or *bla_SHV-18_* was delivered via phagemids and could effectively kill antibiotic-resistant *E. coli* (Citorik et al., 2014). Similarly, a λ phage carrying a CRISPR-Cas3 cascade system and a CRISPR array targeting β-lactam resistance genes successfully eliminated antibiotic resistance plasmids in *E. coli*, restoring its antibiotic sensitivity (Yosef et al., 2015). Selle et al. (2020) demonstrated that ϕCD24-2, a CRISPR-Cas3-armed bacteriophage, effectively eradicated *Clostridioides difficile* in a mouse infection model. CRISPR-Cas-armed bacteriophages have also been utilized to eliminate pathogenic bacteria, such as *Staphylococcus aureus* (Park et al., 2017) and *E. coli* (Gencay et al., 2024), which cause human diseases. However, previous studies have primarily focused on targeting bacteria at the genome or gene level to induce cell death.

In this study, we explored whether temperate λ phages equipped with the Cas12a system could control lysogenic cycle of bacterial cells. Additionally, to precisely distinguish cells with single nucleotide variations (SNVs) in the target genomic DNA, we tested Cas12a-equipped phages carrying truncated CRISPR RNA (crRNA). Using the LacZ phenotype complementation, we evaluated the phage-induced lysis and lysogeny formation in target cells under mixed bacterial culture conditions. Based on our results, we discuss the precise SNV-level control enabled by CRISPR-Cas12a-loaded phages and their potential applications in the microbiome field.

## 2 Materials and methods

### 2.1 Strains and culture conditions

The *E. coli* strains used in this study are listed in Supplementary Table S1. All *E. coli* strains were cultured in Luria-Bertani (LB) broth (LPS Solution, Cat. No. LB-05, Korea) at 30°C or 37°C with shaking at 180 rpm. *E. coli* DH5α was used as the cloning host for crRNA plasmid construction. When necessary, ampicillin (50 μg/mL), kanamycin (25 μg/mL), chloramphenicol (12.5 μg/mL), or spectinomycin (75 μg/mL) was added to the medium. To prepare electrocompetent cells, *E. coli* strains were cultured overnight at either 30°C or 37°C, depending on the experimental requirements: 30°C for strains carrying prophages or temperature-sensitive plasmids (such as pKD46 and pCJH027), and 37°C for strains containing other plasmids. The overnight culture was inoculated into fresh LB broth at 1% of the final volume and cultured until the optical density at 600 nm (OD_600 nm_) reached 0.4. For *E. coli* strains harboring pKD46, L-arabinose was added to a final concentration of 1 mM, followed by an additional 3 h incubation at 30°C. The cells were harvested and washed twice with 10% glycerol. The cells were then resuspended in 10% glycerol at 0.5% (v/v) of the culture volume, aliquoted into 50 μL, and stored at −80°C.

### 2.2 Construction of *galK*-targeting crRNA plasmids

The plasmids used in this study are listed in Supplementary Table S2, and the primers used for plasmid construction are listed in Supplementary Table S3. To construct a crRNA plasmid for LbCas12a, pHL027 (modified from pJYS2_crtYf; Addgene plasmid #85544) carrying the crRNA of FnCas12a was used as a template. Two DNA fragments containing the spectinomycin resistance gene and the *crRNA* gene were amplified using galK_LbCas12a_20_F and galK_LbCas12a_20_R primers (see Supplementary Table S3), and assembled by Gibson assembly using the HiFi DNA Assembly Master Mix (NEB, Cat. No. E2621, USA). The constructed crRNA plasmid for LbCas12a was designated as pCK055. Subsequently, crRNA plasmids targeting the *galK* gene with various lengths of TRS were constructed using pCK055 as a template (Supplementary Figure S1A). The construction of *galK*-targeting crRNA plasmids was confirmed via Sanger sequencing (Supplementary Figure S1B).

### 2.3 Generation of λ *cas12a* phage

The phages used in this study are listed in Supplementary Table S4. To replace the *b2* region of the λ phage genome, which consisted of the *ea59*, *ea31*, and *ea47* genes, with the *Lbcas12a* gene, the P*_rpsL_*-*cas12a*-Cm^R^ cassette was constructed. The *Lbcas12a* fragment was amplified from pCJH027 (a gift from Jennifer Doudna; Addgene plasmid #183074) as the template. The P*_rpsL_* and Cm^R^ fragments were individually amplified from the HL080 template using primer pairs containing homologous sequences required for recombineering. The three purified DNA fragments were ligated using Gibson assembly and subsequently amplified using PCR to create the P*_rpsL_*-*cas12a*-Cm^R^ cassette. The cassette was electroporated into L-arabinose-induced HL081/pKD46 competent cells using a 0.1 cm cuvette (Bio-Rad) under the conditions of 25 μF, 200 *Ω*, and 1.8 kV. Immediately thereafter, 950 μL of SOC medium was added, and the cells were recovered by incubation at 30°C for 2 h with shaking at 180 rpm. The recovered cells were spread onto LB agar plates supplemented with chloramphenicol and incubated at 30°C. The integration of P*_rpsL_*-*cas12a*-Cm^R^ into the *b2* region of the λ prophage genome was confirmed using colony PCR.

### 2.4 Assembly of *cas12a* and *crRNA* genes in the λ phage genome

To insert a *crRNA* in the downstream region of the *cas12a* gene in the CK116 strain, a *cas12a* (532 bp)-P*_J23119_*-*crRNA*-Km^R^ cassette was constructed. A 532-bp fragment of this gene was amplified from CK116 as the template, and the P*_J23119_*-*crRNA* fragment, with a TRS length of 20 nucleotide (nt), was amplified from pCK055. The noncoding region between *cas12a* and P*_J23119_*-*crRNA* and the Km^R^ fragment were separately amplified from HL081. The four DNA fragments were assembled via Gibson assembly and subsequently amplified to generate a single DNA cassette. The *cas12a* (532 bp)-P*_J23119_*-*crRNA*-Km^R^ cassette was electroporated into L-arabinose-induced CK116/pKD46 competent cells. Electroporation was performed as described above. The cells were spread onto LB agar plates supplemented with kanamycin and incubated at 30°C. The integration of the crRNA into CK116 strain was confirmed using colony PCR and Sanger sequencing, and the resulting strain was designated as CK120. To incorporate crRNAs with different TRS lengths into the CK116 strain, *cas12a* (532 bp)-P*_J23119_*-*crRNA*-Km^R^ cassettes, each containing crRNAs with varying TRS lengths, were constructed. These DNA cassettes with overhangs complementary to the *crRNA* gene were amplified using CK120 as a template. The subsequent steps were performed following the procedure used for the construction of CK120. The construction of these phages was confirmed using colony PCR and Sanger sequencing (Supplementary Figure S2).

### 2.5 Incorporation of the *lacZ* gene in the synthetic λ *cas12a-crRNA* phage

To introduce the *lacZ* gene into CK117 (N_23_) and CK124 (N_16_), a *cas12a* (532 bp)-P*_J23119_*-*crRNA*-*lacZ*-Cm^R^ cassette was constructed. The *cas12a* (532 bp)-P*_J23119_*-*crRNA* fragments with different TRS lengths were amplified using CK117 and CK124 as templates. The *lacZ* and Cm^R^ cassettes were amplified from MG1655 and HL080, respectively. The three DNA fragments were assembled via Gibson assembly and amplified to generate the *cas12a* (532 bp)-P*_J23119_*-*crRNA* (N_23_ or N_16_)-*lacZ*-Cm^R^ cassette. The cassette was electroporated into L-arabinose-induced CK117 or CK124 cells harboring the pKD46 plasmid. The recovered cells were spread onto LB agar plates supplemented with chloramphenicol and incubated at 30°C. The location of the *lacZ* gene downstream of the crRNA was confirmed using colony PCR. The PCR primers used for the insertion of *cas12a*, *cas12a-crRNA*, and *cas12a-crRNA-lacZ* into the λ prophage are listed in Supplementary Table S3.

### 2.6 Plaque isolation and phage lysate preparation

Lysogenic cells were initially grown at 30°C and then shifted to 42°C during the log phase. Subsequently, 100 μL of the diluted culture supernatant and 150 μL of phage-free *E. coli* MG1655 cell culture were added to 15 mL of LB top agar containing 5 mM CaCl_2_, 10 mM MgSO_4_, and 0.75% Bacto agar (BD Difco, Cat. No. 214010, USA) and gently mixed. The mixture was then overlaid onto LB agar and incubated at 30°C for 18 h. To obtain phage lysates from the formed plaques, a single phage plaque was added to the *E. coli* MG1655 culture grown in LB broth supplemented with 5 mM CaCl_2_ and 10 mM MgSO_4_. The culture was incubated at 37°C until an OD_600 nm_ of 0.3 was reached, after which it was transferred to 42°C and incubated for 4 h.

### 2.7 Spotting assay

To evaluate the infectivity of λ phages, a spotting assay was performed as follows: 150 μL of overnight culture of *E. coli* host cells was mixed with 15 mL of the LB top agar and overlaid onto an LB agar plate. If needed, X-gal (final 50 μg/mL; Bioneer, Cat. No. C-8002-1, Korea) was added for confirming the β-galactosidase activity caused by infected *lacZ*-incorporated phages. Phage lysates (~ 10^9^ pfu/ml), either undiluted or serially diluted in dilution buffer (100 mM NaCl, 10 mM MgSO_4_, 50 mM Tris–HCl [pH 7.5], 0.01% gelatin), were spotted onto the LB top agar plates and incubated at 30°C for 18 h or at 37°C for 12 h. Clear or turbid spots were observed on a lawn of *E. coli* cells. As negative controls, dilution buffer, fresh LB medium, and supernatant medium collected from Δ*galK* or *galK* WT cell cultures after centrifugation (12,000 rpm, 4°C, 10 min) were spotted on the same plates.

### 2.8 Phage infection in bacterial cultures

Bacterial host cells were grown at 30°C or 37°C in 50 mL of LB broth supplemented with 5 mM CaCl_2_ and 10 mM MgSO_4_ in 125 mL flask with agitation at 180 rpm. When the culture reached an OD_600 nm_ of 0.4, phage lysate (~ 10^9^ pfu/ml) was added at a multiplicity of infection of 0.1. The multiplicity of infection was calculated based on the assumption that 1 mL of culture at an OD_600 nm_ of 1.0 contained 8 × 10^8^ cells. The OD_600 nm_ value was measured using a spectrophotometer (Biochrom Libra S70, Harvard Bioscience, Inc., MA, USA).

For mixed cell cultures, when the OD_600 nm_ reached 0.4, *galK* ^504^A and *galK* WT cells were mixed at an equal ratio. Phage infection and OD_600 nm_ measurements were performed as described above. To assess microbial control using CRISPR-Cas12a-loaded λ phages in the mixed culture, the culture was spread onto MacConkey agar (BD Difco, Cat. No. 281810, USA) plates containing either 0.5% D-galactose (CAS No. 59–23-4) or 0.5% lactose (CAS No. 63–42-3). *galK* ^504^A and *galK* WT cells infected with λ *cas12a galK*-N_16_-*lacZ* were spread on M9 minimal agar (1 × M9 minimal salts, 0.1 mM CaCl_2_, 1 mM MgSO_4_, and 1.5% agar) plates supplemented with 0.5% D-galactose. Ten colonies obtained from the M9 D-galactose plates were randomly selected and streaked on MacConkey agar containing either D-galactose or lactose. The plates were incubated at 30°C for 16 h or 72 h to evaluate phage-mediated bacterial growth and selection.

## 3 Results

### 3.1 Validation of the activity of Cas12a nuclease-loaded λ phage

**Figure 1:**
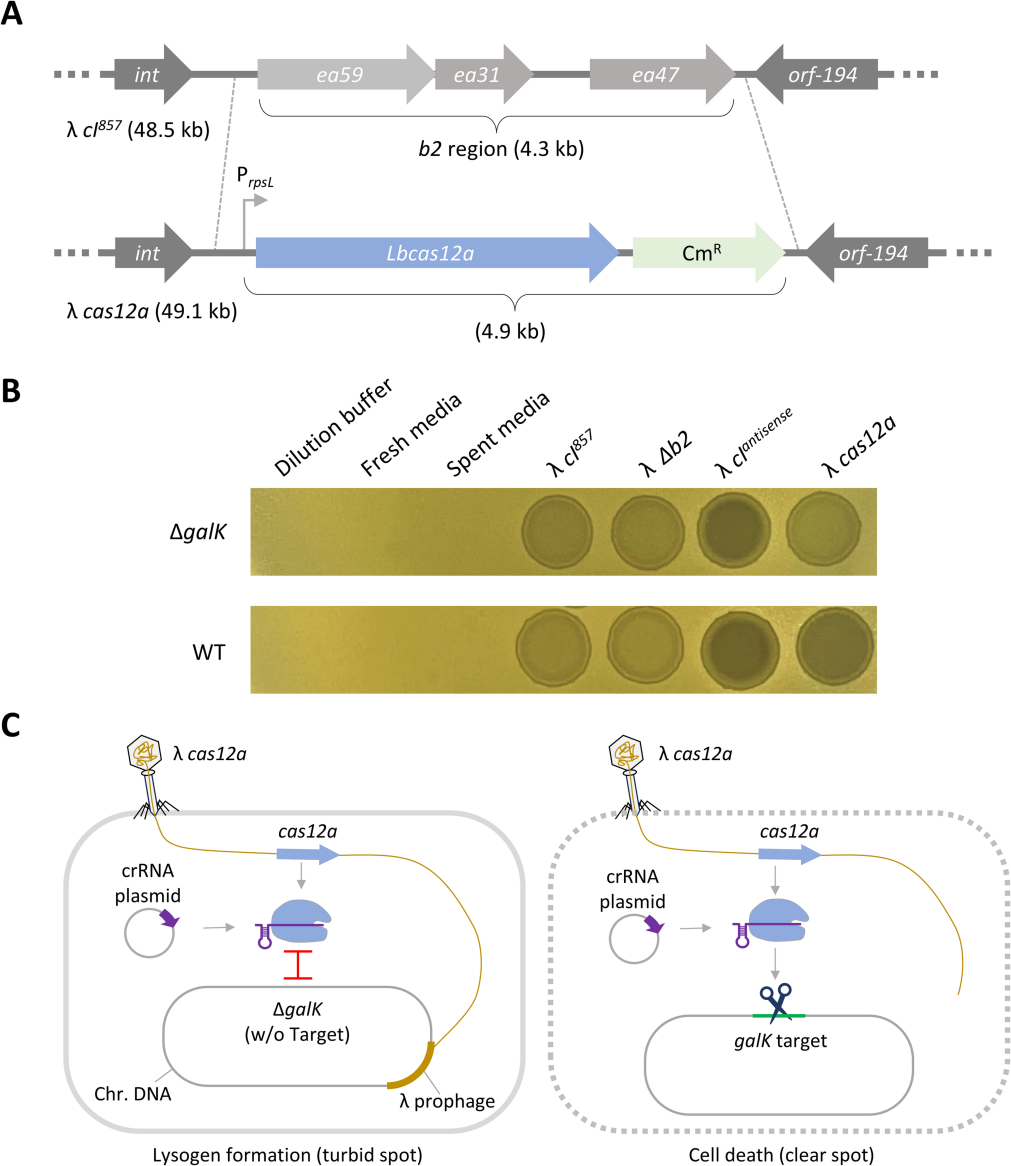
Death of target DNA-containing cells induced by λ phage carrying Cas12a. **(A)** Construction of λ *cas12a* phage. The *cas12a* gene was inserted into the *b2* region of the λ *cI^857^* prophage genome via homologous recombination. **(B)** Spotting assay of various synthetic λ phages on *galK* mutant strains. λ *cI^857^*, λ *Δb2*, λ *cI^antisense^*, and λ *cas12a* were spotted on LB top agar plates with Δ*galK* or *galK* WT cells harboring *galK*-targeting crRNA plasmids. The plates were incubated at 30°C for 18 h. **(C)** Mechanism of cell death induced by λ *cas12a*. λ *cas12a* expresses the Cas12a nuclease and the crRNA plasmid in the host cell generates crRNA targeting the *galK* gene. This leads to the formation of the Cas12a–crRNA complex. If the host genome lacks the *galK* target, the phage remains in the prophage state without inducing cell death. Conversely, the presence of the *galK* target results in cell death.

We constructed the λ *cas12a* phage by inserting the *Lbcas12a* gene into the *b2* region of the λ *cI^857^* phage genome (Figure 1A). λ *cI^857^* is a temperature-sensitive phage with a point mutation in the *cI* gene that enables temperature-dependent genetic switching (Valdez-Cruz et al., 2010). At 28–32°C, the CI repressor suppressed the expression of lytic gene, allowing λ phage to remain in the lysogenic state. However, at temperatures greater than 37°C, the CI repressor became unstable, triggering a switch to the lytic cycle (Villaverde et al., 1993).

To evaluate the activity of the constructed phage, we examined the turbidity of spots formed by it on LB top agar plates inoculated with either Δ*galK* or *galK* WT cells carrying the *galK*-targeting crRNA plasmid (pCK058) at 30°C. Turbid spots were formed on Δ*galK* cells, whereas clear spots were formed on *galK* WT cells (Figure 1B). Clear spots were observed even when phages (~10^9^ pfu/ml) were diluted up to 10^−3^ (Supplementary Figure S3). In addition, to confirm the expression of Cas12a and crRNA in phage-infected lysogenic cells, the *galK*-targeting crRNA plasmid (pCK058) was transformed into λ *cI^857^*- and λ *cas12a*-lysogenic cells (HL051 or CK116), and the number of surviving colonies was compared (Supplementary Figure S4). These results indicate that the Cas12a nuclease and crRNA, expressed from the λ *cas12a* and crRNA plasmid, respectively, formed a Cas12a–crRNA complex capable of effectively recognizing and cleaving the *galK* target, leading to cell death. However, in the absence of the *galK* target, the Cas12a–crRNA complex could not cleave the target DNA, preventing cell death and resulting in the formation of turbid spots (Figure 1C). Additionally, regardless of the target gene, λ *cI^857^* and λ *cI^857^* Δ*b2* formed turbid spots on both Δ*galK* and *galK* WT cells, attributed to their lysogenic cycle. In contrast, λ *cI^antisense^* formed clear spots, indicative of a lytic cycle, because of reduced expression of lysogenic CI repressors in the cells (Figure 1B).

### 3.2 Effect of truncated crRNAs on target recognition and host lysogeny

**Figure 2:**
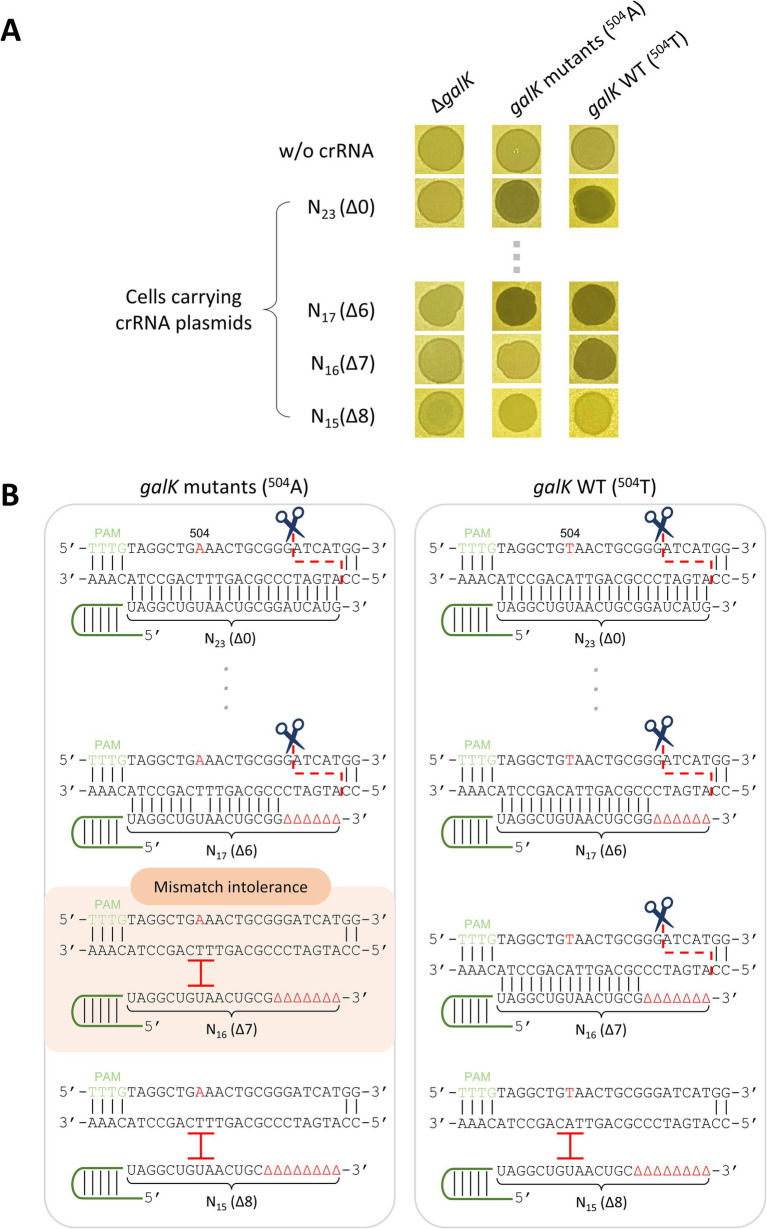
Discrimination of single-nucleotide variations using Cas12a and truncated crRNA. **(A)** Spotting assay of λ *cas12a* on various *galK* strains at 30°C. N_23_ and N_17_-N_15_ represent the length of the target recognition sequence (TRS) in the crRNA, and Δ in the parentheses indicates the number of truncated nucleotides at the 3′-end of the crRNA. **(B)** Mismatch intolerance. “504” represents the 504th nucleotide position in the *galK* gene of the *E. coli* genome. The nucleotides A and T in red represent the variant and original sequences, respectively. The black lines indicate base pairing. The scissors icon and red, dashed line show the position where the Cas12a–crRNA complex recognizes and cleaves the target DNA sequence. The red I indicates that the Cas12a–crRNA complex fails to recognize and cleave the target DNA.

Truncated crRNAs have been reported to enhance the target specificity of Cas12a, allowing it to distinguish SNVs when used as a genome-editing tool (Lee et al., 2022b). Building on this premise, we tested whether truncated crRNAs could differentiate target DNA with single-nucleotide differences and lead to cell death. λ *cas12a* phage lysates were spotted on Δ*galK*, *galK* ^504^A, and *galK* WT cells containing *galK*-targeting crRNA plasmids with different TRS lengths ranging from 23 nt (Δ0) to 15 nt (Δ8). When λ *cas12a* infected cells without crRNA or Δ*galK* cells, turbid spots were formed, indicative of lysogeny. In contrast, clear spots were observed in *galK* ^504^A and *galK* WT cells carrying crRNA plasmids with TRS lengths ranging from 23 nt (Δ0) to 17 nt (Δ6), indicating cell death caused by the cleavage of target genomic DNA by the active Cas12a–crRNA complex (Figure 2A; Supplementary Figure S5). Notably, when using the 16 nt (Δ7) crRNA plasmid, turbid spots were observed in *galK* ^504^A cells, whereas clear spots were observed in *galK* WT cells. For the 15 nt (Δ8) crRNA plasmid, clear spots were not observed in either of these cell genotypes (Figure 2A). These results demonstrated that truncated 16 nt (Δ7) crRNA enabled λ phages carrying Cas12a to distinguish single-nucleotide differences in target DNA, determining host cell death or lysogeny (Figure 2B).

### 3.3 Control of host viability by λ phages armed with truncated crRNAs and Cas12a nuclease

**Figure 3:**
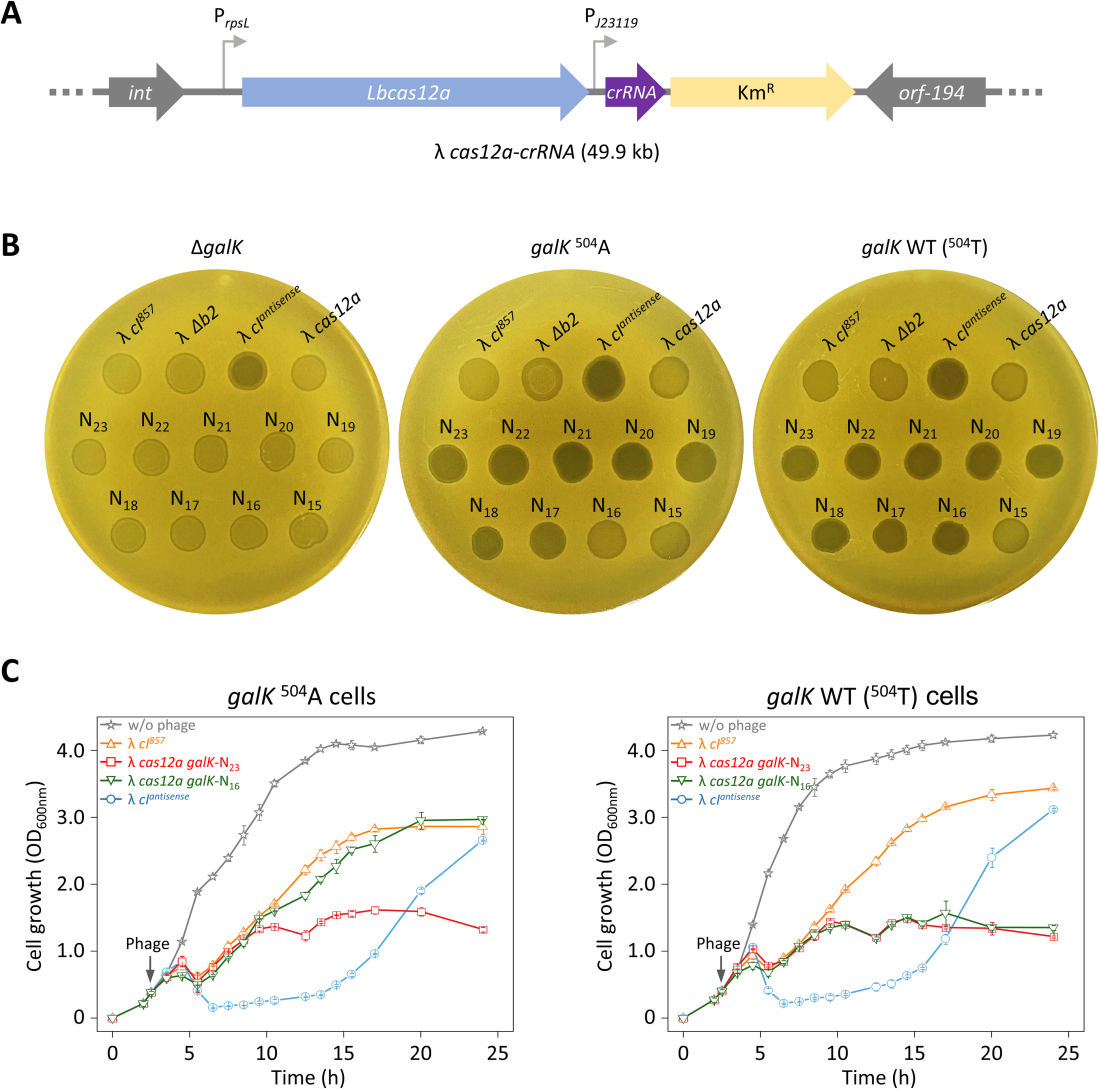
Sequence-specific target recognition and cell death control using λ phage carrying Cas12a and crRNA. **(A)** Construction of λ *cas12a-crRNA* phage. The *cas12a* gene and *crRNA* gene targeting the *galK* gene were inserted into the *b2* region of the λ *cI^857^* prophage genome. **(B)** Spotting assay of various synthetic λ phages including λ *cas12a*-*crRNA* at 30°C. λ *cI^857^*, λ Δ*b2*, λ *cI^antisense^*, λ *cas12a*, and λ *cas12a-crRNA* were spotted on LB top agar plates containing Δ*galK*, *galK* ^504^A, or *galK* WT cells, and incubated at 30°C for 18 h. N_23_-N_15_ represent λ *cas12a-crRNA* with crRNA TRS lengths ranging from 23 nt (Δ0) to 15 nt (Δ8). **(C)** Growth curves of *galK* ^504^A and *galK* WT strains either uninfected or infected with λ *cI^857^*, λ *cI^antisense^*, λ *cas12a galK*-N_23_, and λ *cas12a galK*-N_16_, respectively, at 30°C. The gray arrow indicates the time point of phage infection. OD_600 nm_ values represent the mean obtained from three independent cultures.

We investigated whether the results observed for cells carrying crRNA plasmids were due to multiple copies of the *crRNA* gene or if similar results could be achieved by loading a single copy of crRNA into the λ *cas12a* phage. For this, λ *cas12a-crRNA* phages were constructed by inserting *galK-*targeting crRNAs with various TRS lengths ranging from 23 nt (N_23_) to 15 nt (N_15_) into λ *cas12a* (Figure 3A). Spotting assays were then performed to evaluate whether the Cas12a–crRNA complex expressed from the λ *cas12a-crRNA* phages could control cell death by distinguishing single-nucleotide differences in the target gene within the host genome.

λ *cI^857^*, λ *cI^857^* Δ*b2*, and λ *cas12a* phages produced turbid spots, whereas λ *cI^antisense^* produced clear spots, regardless of the cellular genotype (Δ*galK*, *galK* ^504^A, or *galK* WT) (Figure 3B). As expected, all λ *cas12a*–*crRNA* phages (N_23_–N_15_) formed turbid spots in Δ*galK* cells, as the Cas12a–crRNA complex fails to cleave the genome, thereby preventing cell death and allowing lysogeny, which leads to turbid spot formation. In the case of *galK* ^504^A or *galK* WT cells, clear spots were observed with λ *cas12a*-*crRNA* phages (N_23_ –N_17_). However, λ *cas12a*-*crRNA* phages (N_16_) formed turbid spots in *galK* ^504^A cells, whereas clear spots were observed in *galK* WT cells (Figure 3B). These results indicated that Cas12a and crRNA expressed from λ *cas12a*-*crRNA* form an active complex, which effectively recognizes and cleaves the *galK* target, enabling precise control of target cell death. When incubated at 37°C, the *cI^857^* gene no longer supports the lysogenic cycle, resulting in clear spots on all LB top agar plates regardless of the phage or host cell type (Supplementary Figure S6A).

In view of the results obtained using top agar plates, we investigated whether similar effects would be observed in liquid culture. We monitored the growth of *galK* ^504^A and *galK* WT cells infected with λ *cI^857^*, λ *cI^antisense^*, λ *cas12a galK*-N_23_, or λ *cas12a galK*-N_16_. When infected with lysogenic λ *cI^857^*, cells showed rapid growth recovery after an initial lysis phase between 4 and 6 h. In contrast, when infected with lytic *λ cI^antisense^*, cell growth was suppressed up to 15 h, followed by a period of rapid recovery. The growth patterns associated with λ *cI^857^* and λ *cI^antisense^* were consistent for both the cell types (*galK* ^504^A or *galK* WT). The recovery growth following phage infection could result either from lysogeny or as a consequence of the emergence of phage-insensitive mutants, which is described in the next section.

Cells infected with λ *cas12a galK*-N_23_ showed no significant growth recovery after initial lysis, with OD_600 nm_ remaining stable between 1 and 1.5 for up to 24 h in both *galK* ^504^A and *galK* WT cells at 30°C. In contrast, λ *cas12a galK*-N_16_ exhibited distinct effects depending on the host cell type—in *galK* ^504^A cells, the growth pattern resembled that of cells infected with lysogenic λ *cI^857^*, suggesting that Cas12a failed to recognize and cleave the *galK* ^504^A target; however, in *galK* WT cells, initial growth recovery after lysis was followed by inhibition, closely mimicking the growth suppression pattern observed for λ *cas12a galK*-N_23_-infected cells at 30°C (Figure 3C). At 37°C, Δ*galK*, *galK* ^504^A, and *galK* WT cells infected with various phages, including the lytic λ *cI^antisense^*, exhibited similar growth patterns regardless of the phage type (Supplementary Figure S6B). These results indicated that synthetic phages carrying Cas12a and truncated crRNAs can effectively suppress lysogeny by precisely controlling cell death and distinguishing SNVs within the target DNA.

### 3.4 Removal of genetic variations in mixed bacterial cultures using synthetic phages

**Figure 4:**
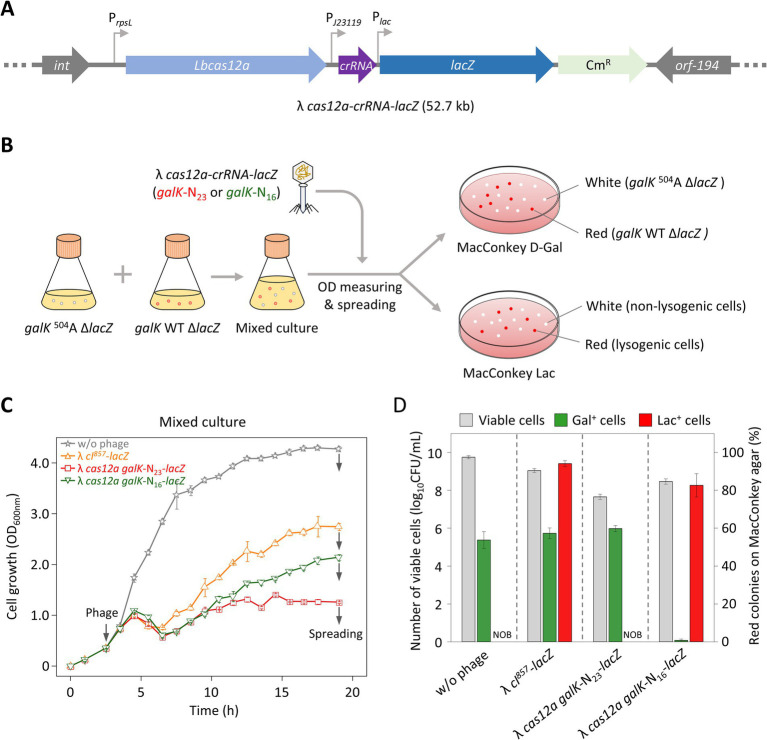
Control of cell death in mixed cell cultures with different *galK* phenotypes using λ phages carrying the CRISPR-Cas system and *lacZ*. **(A)** Construction of the λ *cas12a-crRNA-lacZ* phage. The *cas12a* gene, *galK*-targeting crRNA, and *lacZ* gene were inserted into the *b2* region of the λ *cI^857^* prophage genome. **(B)** Verification of cell death and lysogen formation in *galK* ^504^A Δ*lacZ* and *galK* WT Δ*lacZ* strains on MacConkey agar plates. *galK* ^504^A Δ*lacZ* and *galK* WT Δ*lacZ* cells were cultured to early exponential phase, mixed at equal ratio, and either left uninfected or were infected with the phage. After 19 h of incubation, the cultures were spread on MacConkey D-Gal and MacConkey Lac plates. On the MacConkey D-Gal plate, white and red colonies represent *galK* ^504^A Δ*lacZ* and *galK* WT Δ*lacZ* cells, respectively. On the MacConkey Lac plate, white and red colonies represent non-lysogenic and lysogenic cells, respectively. **(C)** Growth curves of mixed cultures containing *galK* ^504^A Δ*lacZ* and *galK* WT Δ*lacZ* strains, either uninfected or infected with various synthetic λ phages at 30°C. The gray arrows on the left indicate the time point of phage infection, whereas gray arrows on the right indicate the time point of spreading. Each OD_600 nm_ value represents the mean of values for three independent cultures. **(D)** Proportions of *galK* ^504^A Δ*lacZ* and *galK* WT Δ*lacZ* strains, as well as lysogenic cells, observed on MacConkey agar plates. The gray bars represent the total number of viable cells (white colonies + red colonies) observed on the plates. The green bars indicate the proportion of *galK* WT Δ*lacZ* cells among the viable cells, whereas the red bars represent the proportion of lysogenic cells among the viable cells. The data are presented as mean ± standard deviations based on three independent experiments. NOB, Not observed.

As mentioned in the results described above, the regrowth observed after phage infection could have resulted either from lysogeny or from the emergence of phage-insensitive mutants. Previous studies have used colony color changes to identify lysogenic cells (Chaib et al., 2019). However, such changes were not observed with λ phages in our study. To overcome this limitation, we engineered the λ phage genome by inserting the *lacZ* gene, enabling colony color to serve as an indicator of lysogeny. A complementation assay was then performed to confirm that *lacZ* was successfully delivered to Δ*lacZ* target cells during lysogeny. We introduced the *lacZ* gene into λ *cI^857^*, λ *cas12a*-*galK* N_23_, and λ *cas12a*-*galK* N_16_ to make λ *cI^857^*-*lacZ*, λ *cas12a*-*galK* N_23_-*lacZ*, and λ *cas12a*-*galK* N_16_-*lacZ* (Figure 4A). Lysates of these *lacZ*-incorporated phages were spotted on LB top agar containing X-gal and seeded with *galK* WT Δ*lacZ* cells. Blue spots were observed for the *lacZ*-incorporated phages (Supplementary Figure S7A). λ *cas12a*-*galK* N_23_-*lacZ* produced clear spots on both *galK* ^504^A and *galK* WT cells, with no distinction between them. However, λ *cas12a*-*galK* N_16_-*lacZ* formed clear spots only on *galK* WT cells (Supplementary Figure S7B). These results indicated that the presence of the *lacZ* gene did not affect the formation of turbid or clear spots on LB top agar plates containing the target cells.

To investigate whether λ *cas12a galK*-N_23_-*lacZ* or λ *cas12a galK*-N_16_-*lacZ* selectively eliminated target cells while regulating lysogen formation in liquid culture, we treated mixed cell cultures containing strains with a Δ*lacZ* background with *lacZ*-loaded phages. At 19 h after the start of incubation, the culture was spread on MacConkey agar plates containing D-galactose or lactose. The *galK* ^504^A Δ*lacZ* strain, carrying a nonsense mutation in the *galK* gene and unable to metabolize galactose, formed white colonies on MacConkey D-galactose plates, whereas the *galK* WT Δ*lacZ* strain formed red colonies. Furthermore, lysogen host cells infected with *lacZ*-incorporated phages produced red colonies on MacConkey lactose plates, whereas Δ*lacZ* cells that did not acquire the phage genetic material continued to form white colonies (Figure 4B).

Infection with λ *cI^857^*-*lacZ* triggered a rapid recovery of the growth curve in mixed cells following initial cell lysis. In contrast, λ *cas12a galK*-N_23_-*lacZ* infection resulted in only minimal growth recovery. The growth pattern observed with λ *cas12a galK*-N_16_-*lacZ* infection was intermediate between those of λ *cI^857^*-*lacZ* and λ *cas12a galK*-N_23_-*lacZ* infections (Figure 4C). On MacConkey D-galactose plates, infections with either λ *cI^857^*-*lacZ* or λ *cas12a galK*-N_23_-*lacZ* produced a similar ratio of red to white colonies, comparable with that in untreated cultures, indicating that these phages did not distinguish between *galK* ^504^A and *galK* WT cells (Figure 4D). However, λ *cas12a galK*-N_16_-*lacZ* infection resulted in less than 1% of the colonies being red, which indicated that this phage selectively eliminated *galK* WT Δ*lacZ* cells while sparing *galK* ^504^A Δ*lacZ* cells in the mixed culture.

On MacConkey lactose plates, neither the untreated cultures nor those infected with λ *cas12a galK*-N_23_-*lacZ* produced red colonies, indicating that λ *cas12a galK*-N_23_-*lacZ* effectively inhibited lysogen formation. In contrast, infections with λ *cI^857^*-*lacZ* and λ *cas12a galK*-N_16_-*lacZ* resulted in high proportions of red colonies, at 94 and 83%, respectively. To determine whether the red colonies observed on MacConkey galactose plates following λ *cas12a galK*-N_16_-*lacZ* infection were lysogenic, ten colonies randomly selected from M9 D-galactose plates were streaked on either MacConkey D-galactose or MacConkey lactose plates. All red colonies on MacConkey D-galactose plates appeared as white colonies on the MacConkey lactose plates, indicating the *gal*^+^ cells were not lysogenic (Supplementary Figure S8). These results indicated that λ *cI^857^*-*lacZ* forms lysogens regardless of the cell strain, whereas λ *cas12a galK*-N_16_-*lacZ* fails to eliminate *galK* ^504^A cells, allowing them to remain present as lysogens.

## 4 Discussion

Bacteriophage λ is a temperate phage capable of switching between the lytic and lysogenic cycles upon host cell infection. In the lytic cycle, the host cell undergoes lysis, releasing newly assembled phages, whereas in the lysogenic cycle, the phage genome integrates into the host chromosome and is passively replicated (Court et al., 2007). Lysogeny enables prophages to acquire virulence factors and vertically transmit them across generations (Canchaya et al., 2004; Fan and Kan, 2015; Mokrousov, 2009; Naidoo and Zishiri, 2023). Host cells carrying a prophage exhibit superinfection immunity, preventing reinfection by similar phages and potentially limiting bacteriophage-based strategies for microbial control targeting antibiotic-resistant bacteria or pathogens. To overcome this limitation, we engineered a CRISPR-Cas-based λ phage system capable of precise DNA recognition and regulated lysogen formation.

To effectively engineer bacteriophages, it is crucial to identify the nonessential regions of the genome that can be removed and to determine the size limits of foreign genes that can be introduced. The *b2* region of the λ phage is a nonessential gene, and its removal has no impact on the survival or replication of the phage (Casjens and Hendrix, 2015). We, therefore, inserted the *cas12a* gene and crRNAs into the *b2* region of λ *cI^857^* (Figure 1A). Regarding size limits, the λ phage is known to accommodate up to 110% of the wild-type genome length (53.4 kb) while remaining infectious. However, a λ phage with a 53.4 kb genome exhibits a 1,000-fold reduction in infectivity compared with that of the wild-type phage (Nurmemmedov et al., 2012). To address this, we inserted a *cas12a*-*crRNA*-*lacZ* cassette into the λ phage genome (48.5 kb) to create λ *cas12a*-*crRNA*-*lacZ* (52.7 kb), which effectively suppressed lysogen formation and selectively eliminated target cells (Figure 4A).

The CRISPR-Cas system exhibits mismatch tolerance, allowing it to recognize and cleave target DNA even when 1–2 mismatches exist between the gRNA and target DNA (Fu et al., 2013). To overcome this issue, previous studies have successfully performed single-nucleotide editing within the target DNA using truncated gRNA (Lee et al., 2023; Lee et al., 2022b). Therefore, in this study, we designed crRNA plasmids with various TRS lengths to evaluate whether truncated crRNA could distinguish host cells with SNVs and regulate cell lysis (Figure 2A; Supplementary Figure S2). We observed that LbCas12a requires a crRNA with a TRS length of 16 nt (Δ7) to selectively recognize and cleave target DNA at a single-nucleotide resolution, thereby inducing cell lysis. This result is consistent with the minimum TRS length required for FnCas12a to recognize and cleave target DNA (Lee et al., 2022b).

When treated with most phages in this study, cells in liquid culture eventually recovered their growth within 24 h, reaching an OD of 3.0, regardless of whether they were infected by lysogenic λ *cI^857^* or lytic λ *cI^antisense^* (Figure 3C). This growth recovery was likely due to either lysogen formation or the emergence of phage-insensitive mutants. To confirm the actual reason, we constructed *lacZ*-incorporated phages and examined whether the LacZ phenotype was transferred to Δ*lacZ* cells in mixed cultures (Figures 4C,D).

Li et al. reported that the primary mechanism of resistance to phages in *E. coli* is the inability of phages to recognize and bind to specific receptors on the bacterial surface (Li et al., 2019). In particular, a missense mutation in LamB, an essential receptor for λ phage infection, can block the attachment of phages to the host receptor, thereby preventing infection (Charbit et al., 1988; Werts et al., 1994). When λ *cas12a galK*-N_23_-*lacZ* was applied, the viable cell population remained approximately 60% Gal^+^, indicating that the mixture of *galK* ^504^A and *galK* WT cells was maintained and that the cells remained LacZ^−^ (Figure 4D). This suggests that the surviving cells were likely phage-insensitive mutants. We assume that the growth of phage-infected cells was initially inhibited, but it was later restored due to the emergence of phage-resistant mutants, such as those with receptor mutations. In contrast, when cells were treated with λ *cas12a galK*-N_16_-*lacZ*, all surviving cells exhibited the Gal^−^ LacZ^+^ phenotype, indicating the formation of lysogens in *galK* ^504^A cells (Figure 4D). This observation is consistent with previous studies showing that temperate λ phages initially induce lysis upon infecting *E. coli*, but the cell growth subsequently recovers as the lysogen population increases (Maynard et al., 2010; Maynard et al., 2012).

In conclusion, we demonstrated that a temperate phage carrying a Cas12a-truncated crRNA can discriminate host bacteria based on a SNV in the target genome. Guided by the designed truncated crRNA sequence, the synthetic phage precisely modulated lysogeny in the host. Moreover, in a mixed-culture model, the synthetic temperate phage selectively targeted the SNV-containing strain and exhibited lytic activity. These findings offer important insights into the potential application of this strategy as a precision microbiome engineering tool capable of selectively eliminating undesirable bacteria at the SNV level while preserving non-target strains.

## Data Availability

The original contributions presented in the study are included in the article/Supplementary material, further inquiries can be directed to the corresponding author.
