# Supplementary material for: Precision targeting of genetic variations in mixed bacterial cultures using CRISPR-Cas12a-programmed λ phages
Source: Front Microbiol. 2025 Jun 2;16:1575339. doi: 10.3389/fmicb.2025.1575339 (PMC12171280; doi:10.3389/fmicb.2025.1575339)
Supplement: Supplementary file 1 [file Data_Sheet_1.pdf]

Supplementary Materials

Precision Targeting of Genetic Variations in Bacterial Mixed Cultures by CRISPR-Cas12a-Programmed λ Phages

Chan Kyeong Lee, Ho Joung Lee, Song Hee Jeong and Sang Jun Lee*

Department of Systems Biotechnology and Institute of Microbiomics,

Chung-Ang University, Anseong 17546, Republic of Korea

*Corresponding author: sangjlee@cau.ac.kr

Supplementary Table S1. Strains used in this study.

Name	Characteristics	Source/reference
E. coli strains		
DH5α	*fhuA2 lacΔU169 phoA glnV44 Φ80' lacZΔM15 gyrA96 recA1 relA1 endA1 thi-1 hsdR17*	Laboratory stock
MG1655	F^- *ilvG rfb*-50 *rph*-1	Laboratory stock
HK1164	MG1655 *galK* ^{504}A	(Lee et al., 2020)
HL051	MG1655, λ *cI*^{857} lysogen	Laboratory stock
HL062	MG1655, λ *cI*^{857} Δ*b2* lysogen carrying *Ascas12f1*-FRT-Cm^R-FRT	Laboratory stock
HL066	MG1655 Δ*galK*	Laboratory stock
HL080	MG1655 Δ*galK*Δ*xylB*, λ *cI*^{857} Δ*b2* lysogen carrying *Ascas12f1*-FRT-Cm^R-FRT	Laboratory stock
HL081	MG1655 Δ*galK*Δ*xylB*, λ *cI*^{857} Δ*b2* lysogen carrying *Ascas12f1-sgRNA*-FRT-Km^R-FRT (Target: ^{497}TAGGCTGTAACTGCGGGATC^{516} in *galK*)	Laboratory stock
CK116	MG1655 Δ*galK*Δ*xylB*, λ *cI*^{857} Δ*b2* lysogen carrying P_{rpsL}-*Lbcas12a*-FRT-Cm^R-FRT	This study
CK117	MG1655 Δ*galK*Δ*xylB*, λ *cI*^{857} Δ*b2* lysogen carrying P_{rpsL}-*Lbcas12a-crRNA*-FRT-Km^R-FRT (Target: ^{497}TAGGCTGTAACTGCGGGATCATG^{519} in *galK*)	This study
CK118	MG1655 Δ*galK*Δ*xylB*, λ *cI*^{857} Δ*b2* lysogen carrying P_{rpsL}-*Lbcas12a-crRNA*-FRT-Km^R-FRT (Target: ^{497}TAGGCTGTAACTGCGGGATCAT^{518} in *galK*)	This study
CK119	MG1655 Δ*galK*Δ*xylB*, λ *cI*^{857} Δ*b2* lysogen carrying P_{rpsL}-*Lbcas12a-crRNA*-FRT-Km^R-FRT (Target: ^{497}TAGGCTGTAACTGCGGGATCA^{517} in *galK*)	This study
CK120	MG1655 Δ*galK*Δ*xylB*, λ *cI*^{857} Δ*b2* lysogen carrying P_{rpsL}-*Lbcas12a-crRNA*-FRT-Km^R-FRT (Target: ^{497}TAGGCTGTAACTGCGGGATC^{516} in *galK*)	This study
CK121	MG1655 Δ*galK*ΔxylB, λ *cI*^{857} Δ*b2* lysogen carrying P_{rpsL}-*Lbcas12a-crRNA*-FRT-Km^R-FRT (Target: ^{497}TAGGCTGTAACTGCGGGAT^{515} in *galK*)	This study
CK122	MG1655 Δ*galK*Δ*xylB*, λ *cI*^{857} Δ*b2* lysogen carrying P_{rpsL}-*Lbcas12a-crRNA*-FRT-Km^R-FRT (Target: ^{497}TAGGCTGTAACTGCGGGA^{514} in *galK*)	This study
CK123	MG1655 Δ*galK*Δ*xylB*, λ *cI*^{857} Δ*b2* lysogen carrying P_{rpsL}-*Lbcas12a-crRNA*-FRT-Km^R-FRT (Target: ^{497}TAGGCTGTAACTGCGGG^{513} in *galK*)	This study
CK124	MG1655 Δ*galK*Δ*xylB*, λ *cI*^{857} Δ*b2* lysogen carrying P_{rpsL}-*Lbcas12a-crRNA*-FRT-Km^R-FRT (Target: ^{497}TAGGCTGTAACTGCGG^{512} in *galK*)	This study
CK125	MG1655 Δ*galK*Δ*xylB*, λ *cI*^{857} Δ*b2* lysogen carrying P_{rpsL}-*Lbcas12a-crRNA*-FRT-Km^R-FRT (Target: ^{497}TAGGCTGTAACTGCG^{511} in *galK*)	This study
CK155	MG1655 Δ*galK*Δ*xylB*, λ *cI*^{857} Δ*b2* lysogen carrying P_{rpsL}-*Lbcas12a-crRNA-lacZ*-FRT-Cm^R-FRT (Target: ^{497}TAGGCTGTAACTGCGGGATCATG^{519} in *galK*)	This study
CK157	MG1655 Δ*galK*Δ*xylB*, λ *cI*^{857} Δ*b2* lysogen carrying P_{rpsL}-*Lbcas12a-crRNA-lacZ*-FRT-Cm^R-FRT (Target: ^{497}TAGGCTGTAACTGCGG^{512} in *galK*)	This study
CK159	MG1655 *lacZ*::FRT-Km^R-FRT	This study
CK160	MG1655 *galK* ^{504}A *lacZ*::FRT-Km^R-FRT	This study
CK161	MG1655 Δ*galK* *lacZ*::FRT-Km^R-FRT	This study
CK162	MG1655, λ *cI*^{857} Δ*b2* lysogen carrying *lacZ*-FRT-Km^R-FRT	This study

Supplementary Table S2. Plasmids used in this study.

Plasmids	Characteristics	Source/reference
pKD46	pSC101*ori*^{ts}, *araC*, *λ red* genes, Amp^R	(Datsenko and Wanner, 2000)
pCJH027	pSC101 *ori*^{ts}, *lacI*, *LbCas12a*, Km^R	(Huang et al., 2022)
pHL027	pBR322 *ori*, *crRNA* (Target: ^{497}TAGGCTGTCACTGCGGGATC^{516} in *galK*), Sp^R	Laboratory stock
pHL308	pBR322 *ori*, Sp^R	(Kim et al., 2020)
pCK055	pBR322 *ori*, *crRNA* (Target: ^{497}TAGGCTGTAACTGCGGGATC^{516} in *galK*), Sp^R	This study
pCK058	pBR322 *ori*, *crRNA* (Target: ^{497}TAGGCTGTAACTGCGGGATCATG^{519} in *galK*), Sp^R	This study
pCK059	pBR322 *ori*, *crRNA* (Target: ^{497}TAGGCTGTAACTGCGG^{512} in *galK*), Sp^R	This study
pCK060	pBR322 *ori*, *crRNA* (Target: ^{497}TAGGCTGTAACTGCG^{511} in *galK*), Sp^R	This study
pCK061	pBR322 *ori*, *crRNA* (Target: ^{497}TAGGCTGTAACTGCGGGATCAT^{518} in *galK*), Sp^R	This study
pCK062	pBR322 *ori*, *crRNA* (Target: ^{497}TAGGCTGTAACTGCGGGATCA^{517} in *galK*), Sp^R	This study
pCK063	pBR322 *ori*, *crRNA* (Target: ^{497}TAGGCTGTAACTGCGGGAT^{515} in *galK*), Sp^R	This study
pCK064	pBR322 *ori*, *crRNA* (Target: ^{497}TAGGCTGTAACTGCGGGA^{514} in *galK*), Sp^R	This study
pCK065	pBR322 *ori*, *crRNA* (Target: ^{497}TAGGCTGTAACTGCGGG^{513} in *galK*), Sp^R	This study

Supplementary Table S3. Primers used in this study.

Name	Sequence (5'→3')	Description
Sm_ATG_out	GATACTGGGCCGGCAGGCGCTCCATTGCCC	Construction of plasmids expressing *galK*-targeting crRNA
Sm_TAA_out	GCAATGGAGCGCCTGCCGGCCCAGTATCAG	
galK_LbCas12a_20_F	GTTTCAAAGATTAAATAATTTCTACTAAGTGTAGATTAGGCTGTAACTG CGGGATCTTT	Construction of pCK055
galK_LbCas12a_20_R	ATCTACACTTAGTAGAAATTATTTAATCTTTGAAACACTAGTATTATACC TAGGACTGA	
galK_LbCas12a_23_F	TAGGCTGTAACTGCGGGATCATGTTTTTTTGAATTCTCTAGAGTCGACC	Construction of plasmids expressing *galK*-targeting crRNA and λ *cas12a-crRNA* prophages
galK_LbCas12a_23_R	CATGATCCCGCAGTTACAGCCTAATCTACACTTAGTAGAAATTATTTAA	
galK_LbCas12a_22_F	TAGGCTGTAACTGCGGGATCATTTTTTTTGAATTCTCTAGAGTCGACC	
galK_LbCas12a_22_R	ATGATCCCGCAGTTACAGCCTAATCTACACTTAGTAGAAATTATTTAA	
galK_LbCas12a_21_F	TAGGCTGTAACTGCGGGATCATTTTTTTGAATTCTCTAGAGTCGACC	
galK_LbCas12a_21_R	TGATCCCGCAGTTACAGCCTAATCTACACTTAGTAGAAATTATTTAA	
galK_LbCas12a_19_F	ATTAGGCTGTAACTGCGGGATTTTTTTTGAATTCTCTAGAGTCGACC	
galK_LbCas12a_19_R	ATCCCGCAGTTACAGCCTAATCTACACTTAGTAGAAATTATTTAA	
galK_LbCas12a_18_F	GATTAGGCTGTAACTGCGGGATTTTTTTGAATTCTCTAGAGTCGACC	
galK_LbCas12a_18_R	TCCCGCAGTTACAGCCTAATCTACACTTAGTAGAAATTATTTAA	
galK_LbCas12a_17_F	AGATTAGGCTGTAACTGCGGGTTTTTTTGAATTCTCTAGAGTCGACC	
galK_LbCas12a_17_R	CCCGCAGTTACAGCCTAATCTACACTTAGTAGAAATTATTTAA	
galK_LbCas12a_16_F	TAGATTAGGCTGTAACTGCGGTTTTTTTGAATTCTCTAGAGTCGACC	
galK_LbCas12a_16_R	CCGCAGTTACAGCCTAATCTACACTTAGTAGAAATTATTTAA	
galK_LbCas12a_15_F	GTAGATTAGGCTGTAACTGCGTTTTTTTGAATTCTCTAGAGTCGACC	
galK_LbCas12a_15_R	CGCAGTTACAGCCTAATCTACACTTAGTAGAAATTATTTAA	
orf194_TAAOut	GAGGCATCGTGGCATCTCGTTGAAGACC	Construction of all synthetic λ prophages
b2_F	GAGAAGCACAAAGCCTCGCAATCCAGTG	Construction of CK116 strain
PrpsL_LbCas12a_R	AACTTCTCCAGTTTGCTCATTAAATAGCTCCTGGTTTTAGCTTTTG	
PrpsL_LbCas12a_F	CTAAAACCAGGAGCTATTTAATGAGCAAACTGGAGAAGTTCACGAA	
LbCas12a_CmR_F	CGTGAAACATTAAGGGATCCGTATACCGTGTAGGC	
LbCas12a_CmR_R	TACGGATCCCTTAATGTTTCACGCTGGTCTGCGCA	
LbCpf1_3156_F	CTCGCGTACGGATGCCGATTATATC	Construction of λ *cas12a-crRNA* and λ *cas12a-crRNA-lacZ* prophages
LbCas12a_ea47_ol_R	TTTTTTCTTCGTTTTCTCTATTAATGTTTCACGCTGGTCTGCGCAT	Construction of CK120 strain
LbCas12a_ea47_ol_F	AGACCAGCGTGAAACATTAATAGAGAAAACGAAGAAAAAAAACCGA	
sgRNA_sacI_1R	CTGACTCGCTGCGCTCGAGCTCAAAAAAAAAATTGCTTTTAAGAC	
sgRNA_sacI_1F	GCAATTTTTTTTTTGAGCTCGAGCGCAGCGAGTCAGTGAGCGAGG	
CmR_sgRNA_N2_R	CCCGGAATCCAGCCTACACGGTATACGGATCCCAAACTTATCATCCCCT TTTGCTTATG	
sgRNA_CmR_N2_F	GCAAAAGGGGATGATAAGTTTGGGATCCGTATACCGTGTAGGCTGGATT CCGGGGATCC	
crRNA_plac_R	GGATGATAAGTTTGCGCAACGCAATTAATGTGAG	Construction of CK155, CK157 strains
crRNA_plac_F	GGATGATAAGTTTGCGCAACGCAATTAATGTGAG	
lacZ_CmR_R	GGTATACGGATCCCTTATTTTTGACACCAGACCAACTGG	
lacZ_CmR_F	CTGGTGTCAAAAATAAGGGATCCGTATACCGTGTAGGCTGG	
lacZ_KmR_ol_F	CGTATGTTGTGTGGAATTGTGAGCGGATAACAATTTCACACAGGAAACA GCTATTCCGGGGATCCGTCGACC	Construction of CK159-CK161 strains
lacZ_KmR_ol_R	CATAATGGATTTCCTTACGCGAAATACGGGCAGACATGGCCTGCCCGGT TATTATGTAGGCTGGAGCTGCTTCG	

Supplementary Table S4. Phages used in this study.

Name	Characteristics	Source/reference
λ *cI*^{antisense}	λ *cI*^{antisense}	(Lee et al., 2022)
λ *cI*^{857}	λ *cI*^{857}	Laboratory stock
λ *cI*^{857} Δ*b2*	λ *cI*^{857} Δ*b2* carrying FRT-Cm^{R}-FRT	Laboratory stock
λ *cas12a*	λ *cI*^{857} Δ*b2* carrying P_{rpsL}-*Lbcas12a*-FRT-Cm^{R}-FRT	This study
λ *cas12a galK*-N_{23}	λ *cI*^{857} Δ*b2* carrying P_{rpsL}-*Lbcas12a-crRNA*-FRT-Km^{R}-FRT (Target: ^{497}TAGGCTGTAACTGCGGGATCATG^{519} in *galK*)	This study
λ *cas12a galK*-N_{22}	λ *cI*^{857} Δ*b2* carrying P_{rpsL}-*Lbcas12a-crRNA*-FRT-Km^{R}-FRT (Target: ^{497}TAGGCTGTAACTGCGGGATCAT^{518} in *galK*)	This study
λ *cas12a galK*-N_{21}	λ *cI*^{857} Δ*b2* carrying P_{rpsL}-*Lbcas12a-crRNA*-FRT-Km^{R}-FRT (Target: ^{497}TAGGCTGTAACTGCGGGATCA^{517} in *galK*)	This study
λ *cas12a galK*-N_{20}	λ *cI*^{857} Δ*b2* carrying P_{rpsL}-*Lbcas12a-crRNA*-FRT-Km^{R}-FRT (Target: ^{497}TAGGCTGTAACTGCGGGATC^{516} in *galK*)	This study
λ *cas12a galK*-N_{19}	λ *cI*^{857} Δ*b2* carrying P_{rpsL}-*Lbcas12a-crRNA*-FRT-Km^{R}-FRT (Target: ^{497}TAGGCTGTAACTGCGGGAT^{515} in *galK*)	This study
λ *cas12a galK*-N_{18}	λ *cI*^{857} Δ*b2* carrying P_{rpsL}-*Lbcas12a-crRNA*-FRT-Km^{R}-FRT (Target: ^{497}TAGGCTGTAACTGCGGGA^{514} in *galK*)	This study
λ *cas12a galK*-N_{17}	λ *cI*^{857} Δ*b2* carrying P_{rpsL}-*Lbcas12a-crRNA*-FRT-Km^{R}-FRT (Target: ^{497}TAGGCTGTAACTGCGGG^{513} in *galK*)	This study
λ *cas12a galK*-N_{16}	λ *cI*^{857} Δ*b2* carrying P_{rpsL}-*Lbcas12a-crRNA*-FRT-Km^{R}-FRT (Target: ^{497}TAGGCTGTAACTGCGG^{512} in *galK*)	This study
λ *cas12a galK*-N_{15}	λ *cI*^{857} Δ*b2* carrying P_{rpsL}-*Lbcas12a-crRNA*-FRT-Km^{R}-FRT (Target: ^{497}TAGGCTGTAACTGCG^{511} in *galK*)	This study
λ *cI*^{857}-*lacZ*	λ *cI*^{857} Δ*b2* carrying *lacZ*-FRT-Km^{R}-FRT	This study
λ *cas12a galK*-N_{23}-*lacZ*	λ *cI*^{857} Δ*b2* carrying P_{rpsL}-*Lbcas12a-crRNA-lacZ*-FRT-Cm^{R}-FRT (Target: ^{497}TAGGCTGTAACTGCGGGATCATG^{519} in *galK*)	This study
λ *cas12a galK*-N_{16}-*lacZ*	λ *cI*^{857} Δ*b2* carrying P_{rpsL}-*Lbcas12a-crRNA-lacZ*-FRT-Cm^{R}-FRT (Target: ^{497}TAGGCTGTAACTGCGG^{512} in *galK*)	This study

References

1. Datsenko, K.A., and Wanner, B.L. (2000). One-step inactivation of chromosomal genes in *Escherichia coli* K-12 using PCR products. *Proc. Natl. Acad. Sci. U. S. A.*, 97(12), 6640-6645.
2. Huang, C.J., Adler, B.A., and Doudna, J.A. (2022). A naturally DNase-free CRISPR-Cas12c enzyme silences gene expression. *Mol. Cell*., 82(11), 2148-2160 e2144.
3. Kim, B., Kim, H.J., and Lee, S.J. (2020). Regulation of Microbial Metabolic Rates Using CRISPR Interference With Expanded PAM Sequences. *Front. Microbiol*., 11, 282.
4. Lee, H.J., Kim, H.J., and Lee, S.J. (2020). CRISPR-Cas9-mediated pinpoint microbial genome editing aided by target-mismatched sgRNAs. *Genome Res.*, 30(5), 768-775.
5. Lee, H.J., Kim, H.J., and Lee, S.J. (2022). Control of lambda Lysogenic *Escherichia coli* Cells by Synthetic lambda Phage Carrying cIantisense. *ACS Synth. Biol.* 11(11), 3829-3835.

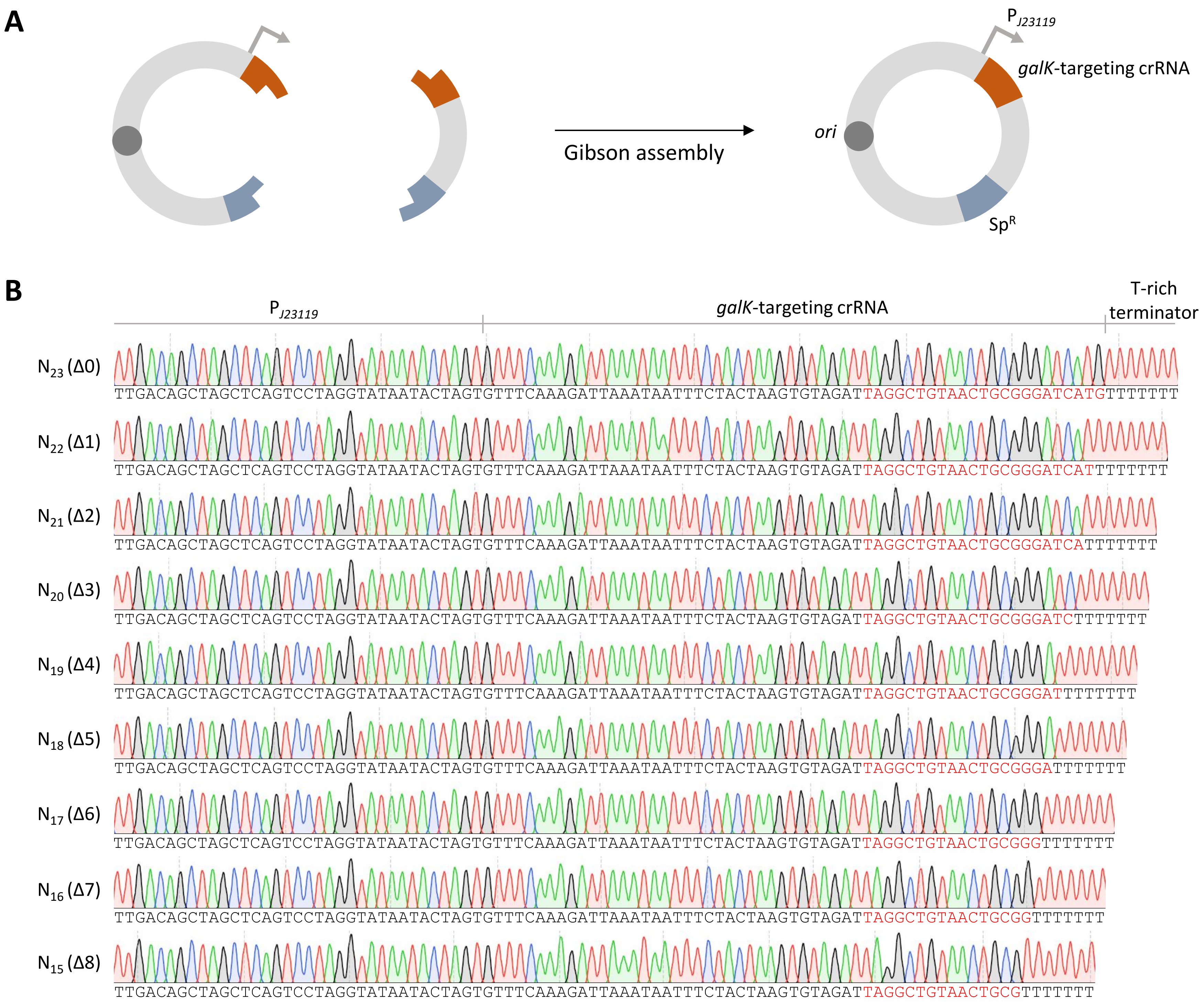

Supplementary Figure S1. Plasmids expressing *galK*-targeting crRNA. **(A)** Construction of *galK*-targeting crRNA plasmids. The *galK*-targeting crRNA plasmids were constructed using Gibson assembly with two DNA fragments containing overhangs, which consisted of a crRNA scaffold and a spectinomycin resistance gene. **(B)** Sanger sequencing results of *galK*-targeting crRNA plasmids. N_{23}-N_{15} represent the lengths of the target recognition sequence (TRS) in the crRNA, highlighted in red. The symbol Δ indicates the number of nucleotides truncated from the 3′ end of the crRNA.

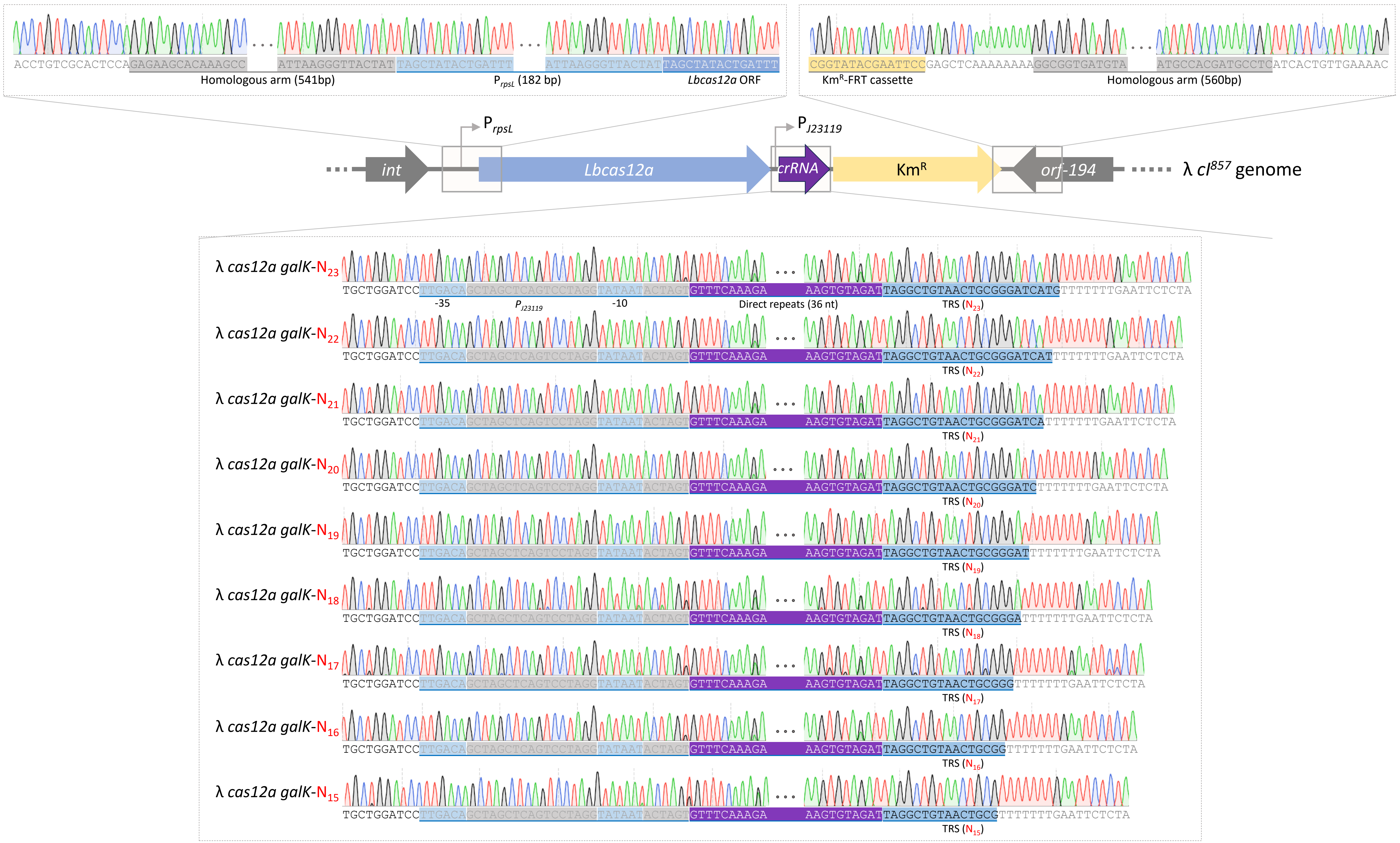

Supplementary Figure S2. Sanger sequencing of λ *cas12a-crRNA*. The *cas12a* gene and each *crRNA* gene with a TRS ranging from 23 nt (Δ0) to 15 nt (Δ8) were inserted into the *b2* region of the λ *cI*^{857} prophage genome.

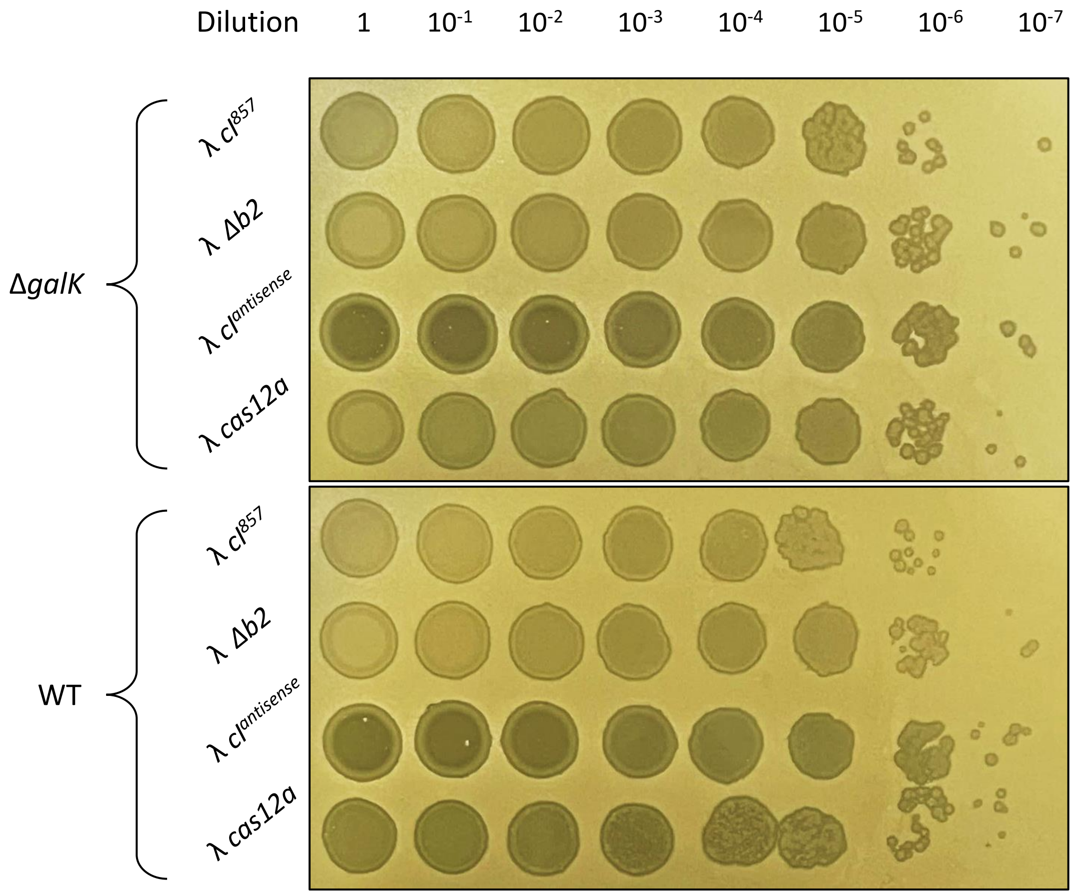

Supplementary Figure S3. Spotting assay of serially diluted synthetic λ phages. λ *cI*^{857}, λ *Δb2*, λ *cI*^{antisense}, and λ *cas12a* phages were serially diluted and spotted onto LB top agar plates seeded with either Δ*galK* or *galK* WT cells harboring *galK*-targeting crRNA plasmids. The plates were incubated at 30°C for 18 h.

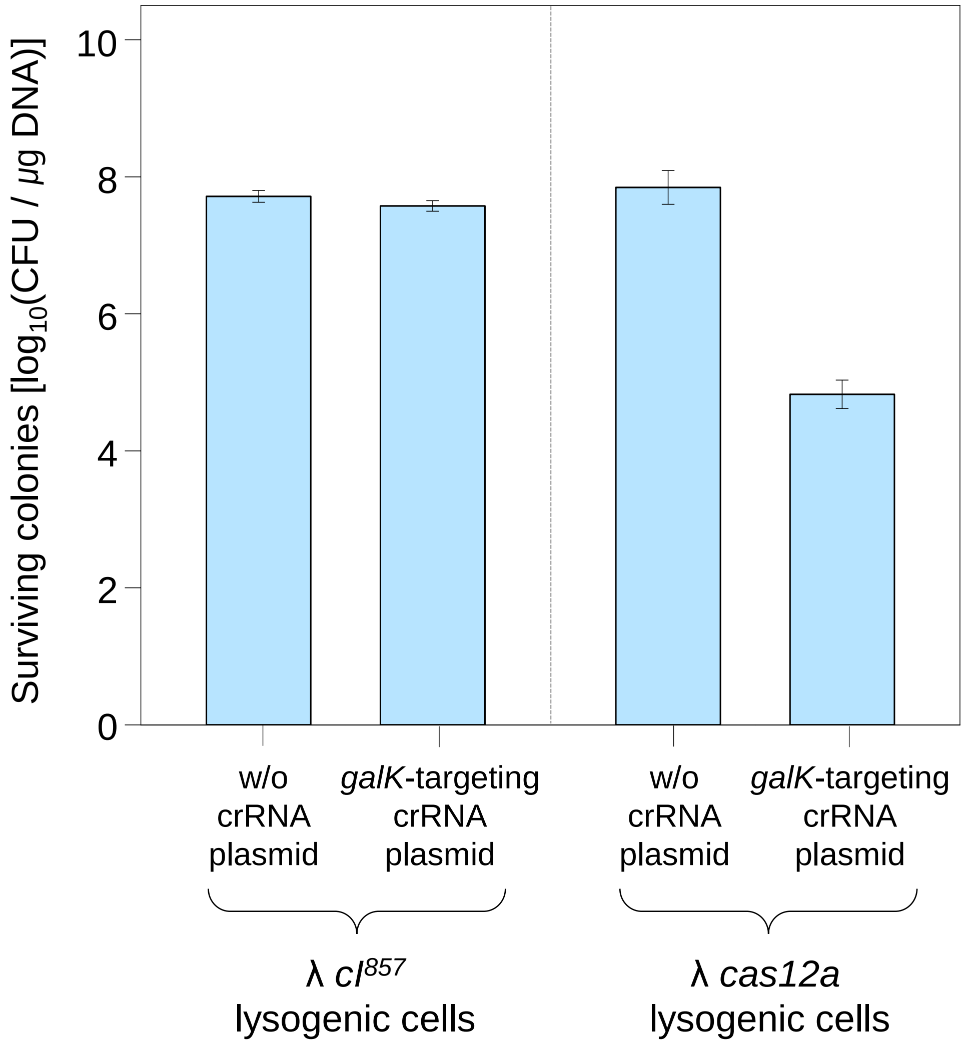

Supplementary Figure S4. Transformation efficiency of *galK*-targeting crRNA plasmids in λ cI^{857}- and λ *cas12a*-lysogenic cells. The number of surviving colonies reflects whether the *cas12a* gene integrated in the λ *cas12a*-lysogenic genome and the *galK*-targeting crRNA are properly expressed, thereby enabling formation of an active Cas12a-crRNA complex that cleaves the target *galK* locus and induces cell death.

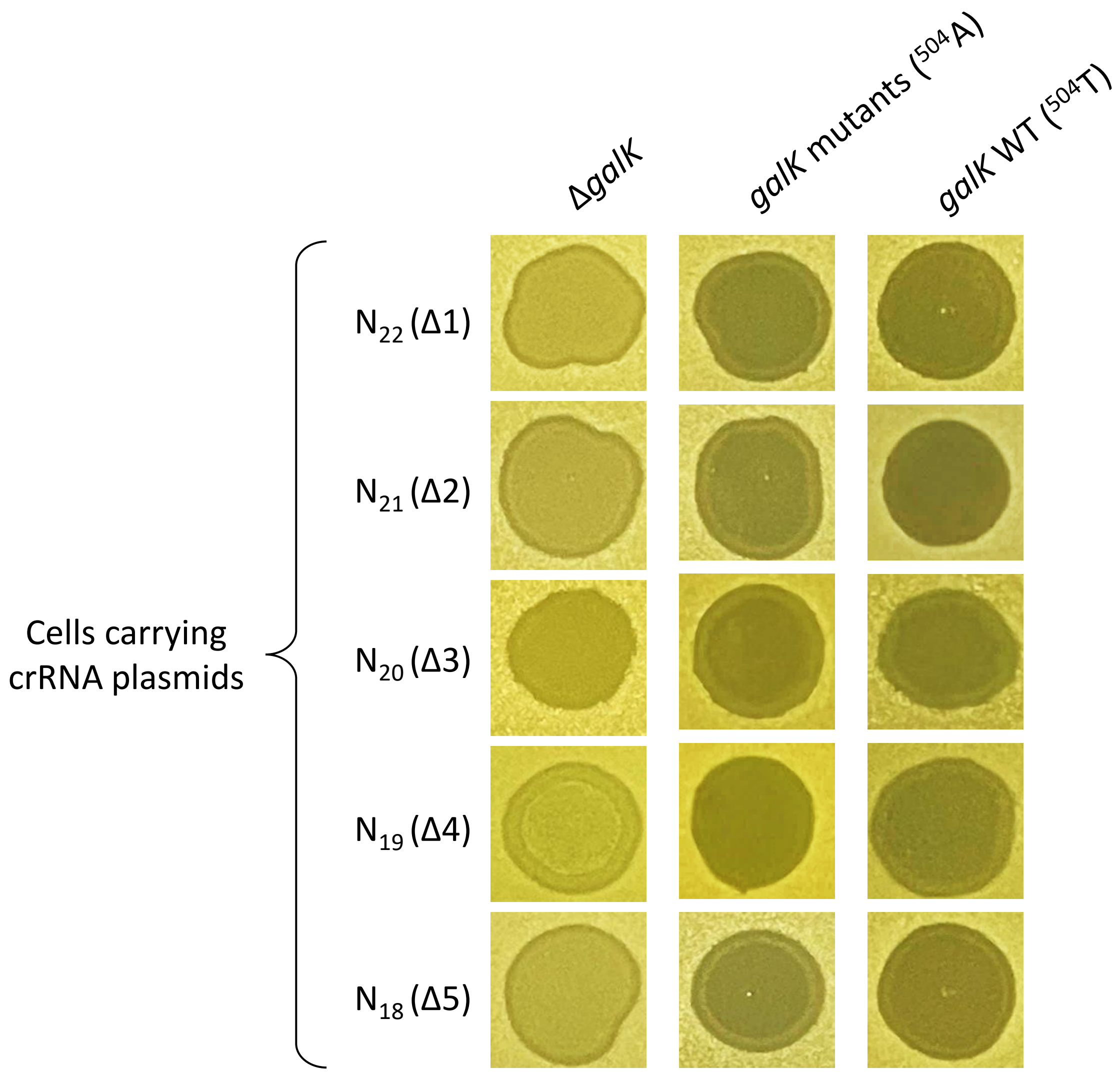

Supplementary Figure S5. Cell-killing effect of λ *cas12a* in various *galK* strains harboring 3′-truncated crRNA plasmids. λ *cas12a* was spotted onto LB top agar plates containing Δ*galK*, *galK* ^{504}A, or *galK* WT cells harboring crRNA plasmids with TRS ranging from 22 nt (Δ1) to 18 nt (Δ5). The plates were incubated at 30°C for 18 h.

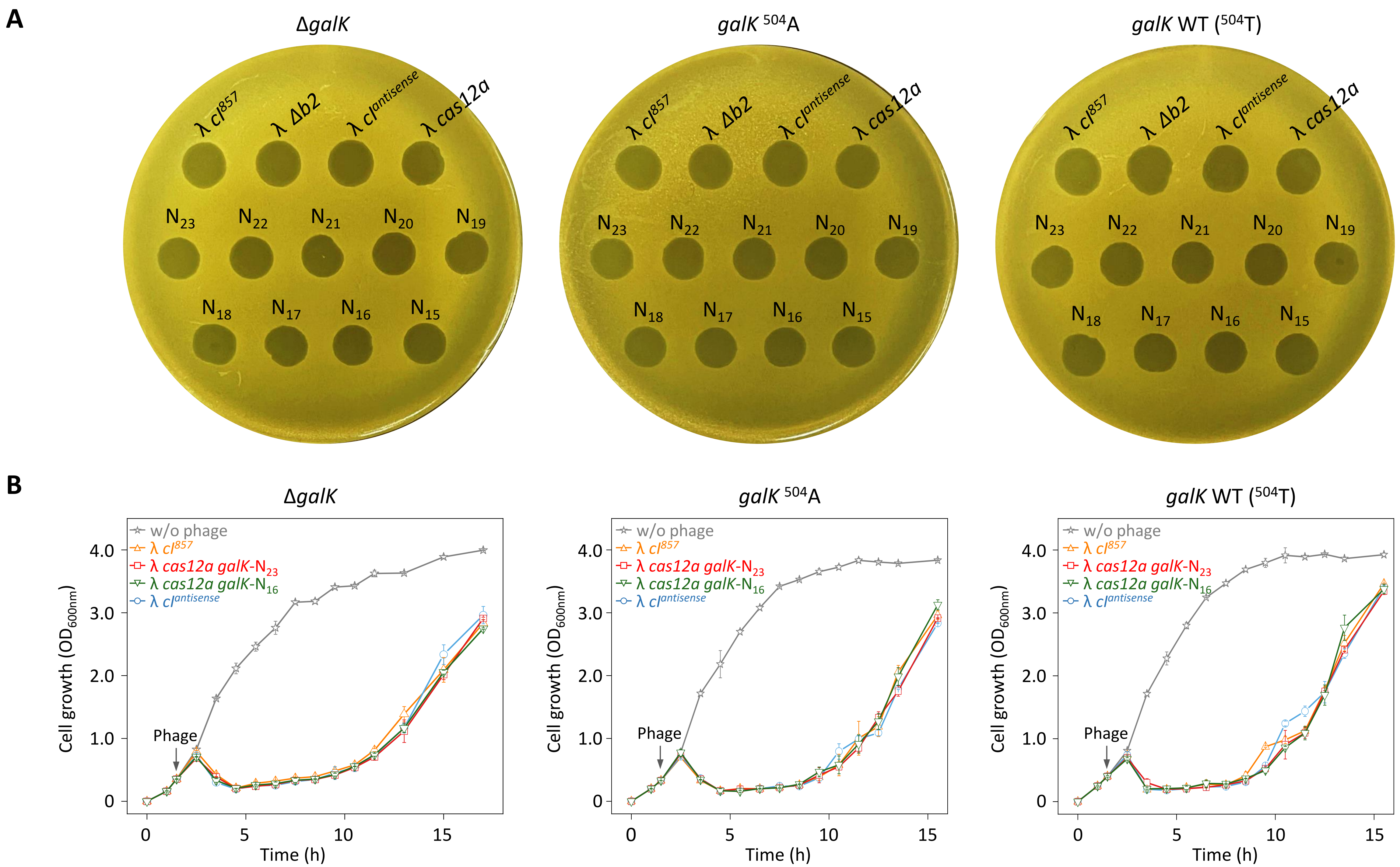

Supplementary Figure S6. Growth curves of various *E. coli* cells infected with engineered λ phages at 37°C. **(A)** Spotting assay of λ *cas12a-crRNA* on LB top agar plates containing various *galK* strains at 37°C. λ cI^{857}, λ Δ*b2*, λ $cI^{antisense}$, λ *cas12a*, and λ *cas12a-crRNA* were spotted onto LB top agar plates containing Δ*galK*, *galK* ^{504}A, or *galK* WT cells, followed by incubation at 37°C for 12 h. N_{23}–N_{15} represent λ *cas12a*-crRNA with TRS lengths ranging from 23 nt (Δ0) to 15 nt (Δ8). **(B)** Growth curves of Δ*galK*, *galK* ^{504}A, and *galK* WT strains either uninfected or infected with λ cI^{857}, λ $cI^{antisense}$, λ *cas12a galK*-N_{23}, or λ *cas12a galK*-N_{16} at 37°C. The gray arrow indicates the time point of phage infection. $OD_{600\ nm}$ values represent the mean obtained from three independent cultures.

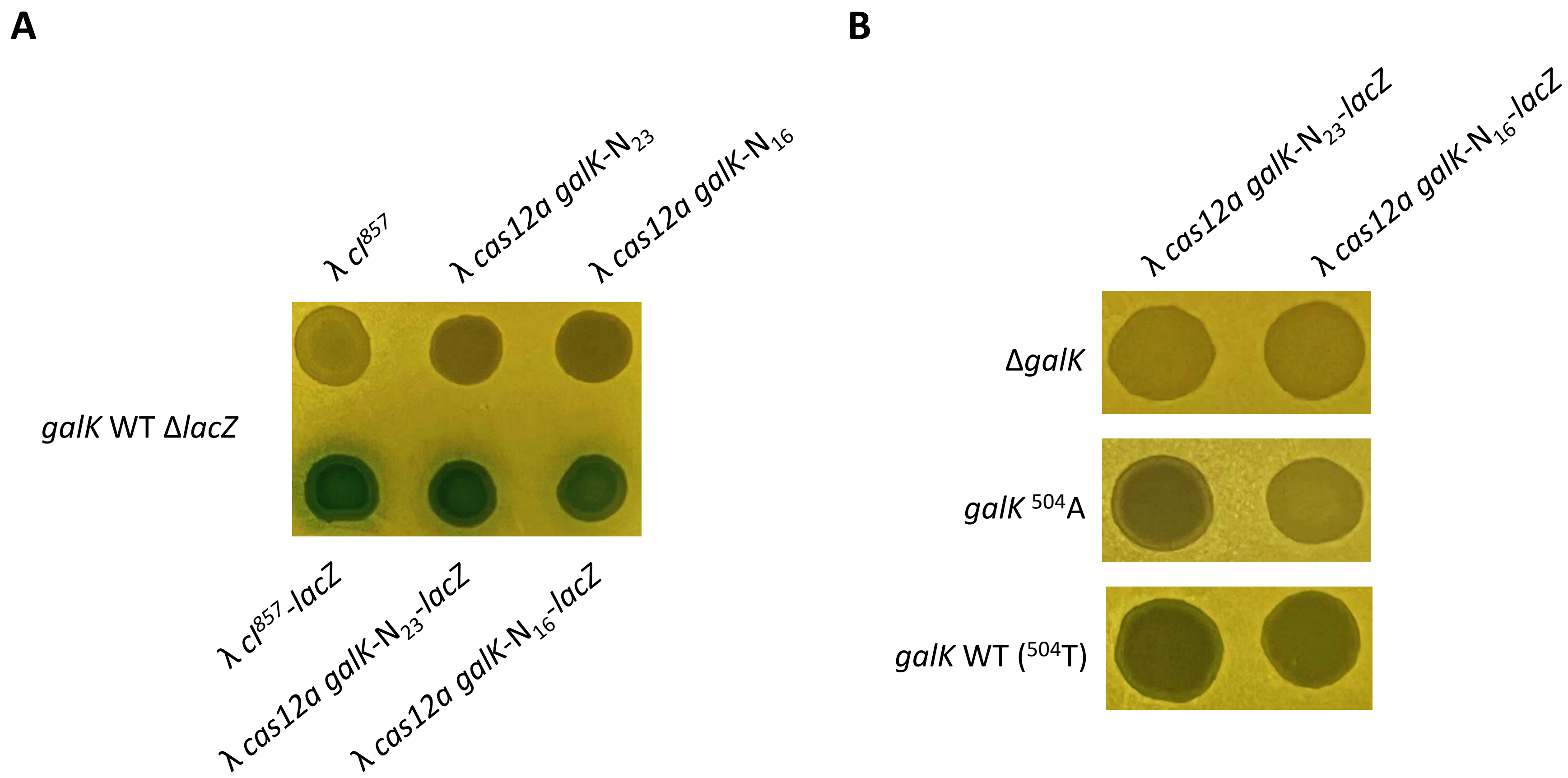

Supplementary Figure S7. Spotting assay of engineered λ phages carrying the *lacZ* gene at 30°C. **(A)** Verification of *lacZ* expression in engineered λ phages carrying the *lacZ* gene. Various engineered λ phages were spotted onto LB top agar plates containing *galK* WT Δ*lacZ* cells and X-gal, followed by incubation at 30°C for 18 h. **(B)** Spotting assay of λ *cas12a galK*-N_{23}-*lacZ* and λ *cas12a galK*-N_{16}-*lacZ* in various *galK* mutant strains. λ *cas12a galK*-N_{23}-*lacZ* and λ *cas12a galK*-N_{16}-*lacZ* were spotted onto LB top agar plates containing Δ*galK*, *galK* ^{504}A, or *galK* WT cells, followed by incubation at 30°C for 18 h.

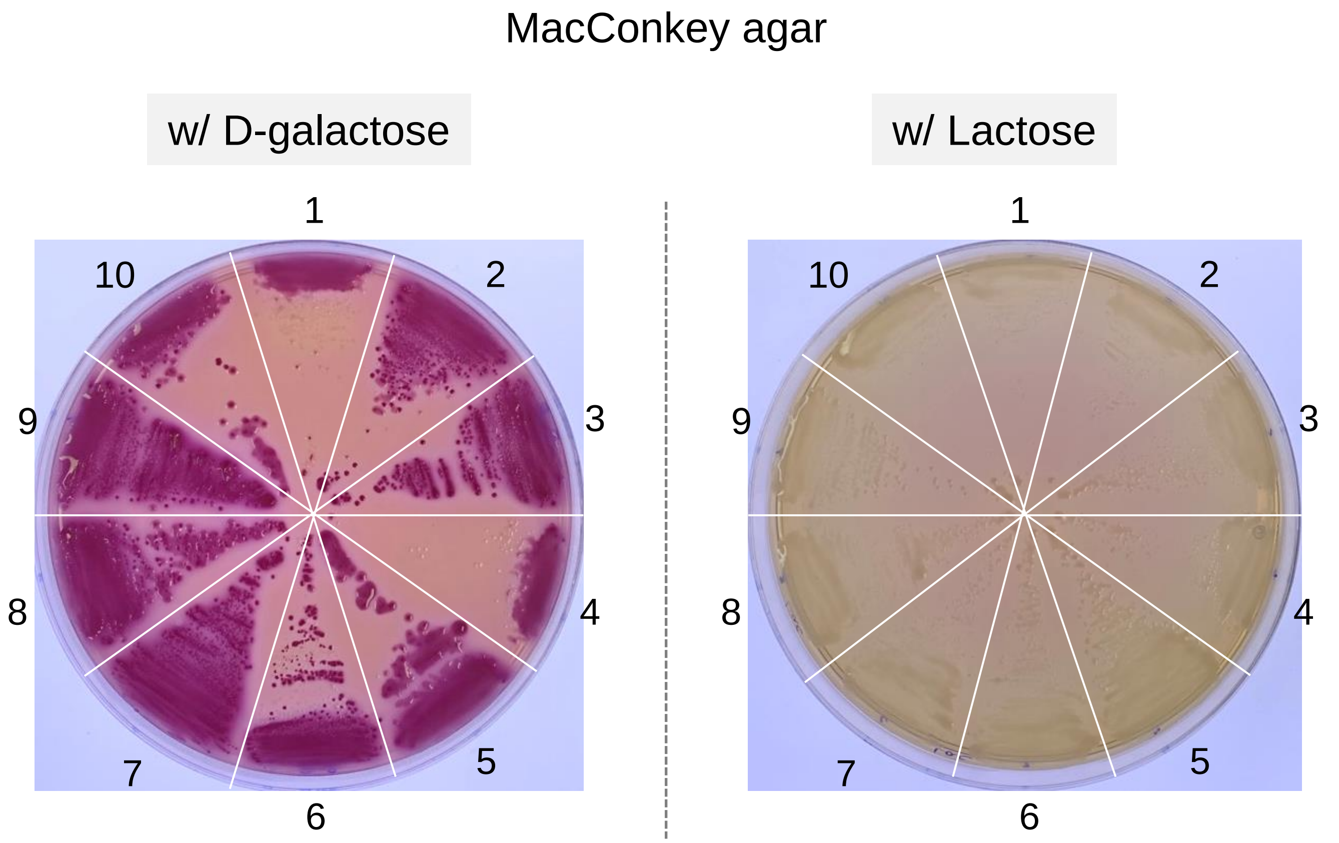

Supplementary Figure S8. Phenotypic differentiation of phage-Infected mixed cultures using MacConkey indicator plates. Mixed cultures of *galK* ^{504}A Δ*lacZ* and *galK*$^{+}$ Δ*lacZ* strains were infected with λ *cas12a galK*-N_{16}-*lacZ* phages and plated on M9 D-Gal medium. After incubation at 30°C for 72 h, ten colonies were randomly selected and streaked onto MacConkey D-Gal or MacConkey Lac plates. On MacConkey D-Gal plates, white and red colonies represent *galK* ^{504}A Δ*lacZ* and *galK*$^{+}$ Δ*lacZ* cells, respectively. On MacConkey Lac plates, white colonies indicate non-lysogenic cells, while red colonies represent lysogenic cells.
